# Value of Vaccination

**Published:** 1893-12

**Authors:** 


### VALUE OF VACCINATION.

Under this title *The Daily Eagle*, of Union City, Indiana, is making a crusade against vaccination. In an article published in the number for September 20th it gives some statistics. Here is an extract from the article mentioned:

"Let us consider the matter by presenting some of the claims and some of the facts. In the first place: Does vaccination *prevent* small-pox?

"If it does, why do vaccinated people take it? They do take it, don't they? We think they do. In the French army every recruit is vaccinated when he enlists and it must take well and good before he enters the service. This is certainly a fair test. Well, in 1870, during the Franco-Prussian war, there were 23,469 cases of small-pox in the French army, all of them vaccinated and most of them re-vaccinated!

"Take the hospital reports. The Deptford (England) Hospital Report for 1879 gives the following: Vaccinated patients, with one mark, 317; two marks, 384; three marks, 447. The

Homerton Hospital for the years 1871 to 1877 gives the number of vaccinated patients with one mark at 1,042; two marks, 1,259; three or more, 1,261. The Dulham Hospital Report ot 1878 gives: patients with one mark, 149; two marks, 156; three or more, 202. The Metropolitan Hospital Report, 1870–2, gives: patients with one mark, 1,124; two marks, 1,722; three marks or more, 1,677."

